# Enantioselective Total Synthesis of (+)-Lyngbyabellin M

**DOI:** 10.3390/md13063309

**Published:** 2015-05-27

**Authors:** Rodrigo V. Pirovani, Gilmar A. Brito, Rosimeire C. Barcelos, Ronaldo A. Pilli

**Affiliations:** Institute of Chemistry, University of Campinas (UNICAMP), P.O. Box 6154, CEP 13083-970 Campinas, São Paulo, Brazil; E-Mails: vezula@gmail.com (R.V.P.); gilmarbrj@gmail.com (G.A.B.); rosicbarcelos@gmail.com (R.C.B.)

**Keywords:** lyngbyabellin M, total synthesis, chiral thiazoles, stereoselective *anti* aldol

## Abstract

Lyngbyabellin M is a non-ribosomal peptide synthetase/polyketide synthase derived metabolite isolated from the cyanobacterium *M. bouillonii* displaying thiazole rings and a distinct chlorinated octanoic acid chain. Its absolute configuration was proposed based on the comparison of its spectroscopic data with those of other representatives of this family of marine natural products, as well as degradation and derivatization studies. Here the first total synthesis of (+)-lyngbyabellin M is described based on the coupling of three key intermediates: two chiral thiazole moieties and an *anti* hydroxycarboxylic acid prepared stereoselectively via a boron enolate mediated aldol reaction directed by Masamune’s chiral auxiliary.

## 1. Introduction

Marine natural products represent an untapped source of structurally diverse and biologically relevant molecules and have inspired novel compounds that have entered clinical trials, and some have been approved by the Food and Drug Administration (USA). The widespread use of genome sequencing is expected to contribute to increase the number of marine natural products in the medicinal chemistry pipeline [1].

Cyanobacteria are a rich source of biologically active natural compounds, and over the last years it has been recognized that many of the natural products isolated from marine animals such as sponges, tunicates and bryozoans are, in fact, produced by symbiotic cyanobacteria. The majority of cyanobacteria metabolites are products from either the non-ribosomal polypeptide (NRP) or the mixed polyketide-non-ribosomal polypeptide biosynthetic pathways [2]. A particular pharmacological feature of the marine cyanobacterial compounds is their ability to act as microtubule and actin disruptors with dolastatins 10 and 15 being two of the earliest representatives [3].

The findings that many marine natural products structurally resembling those produced by terrestrian microorganisms could be produced by marine bacteria or fungi led to the challenge of providing sufficient amounts of the natural products in order to unravel their biological properties, not only because of their small natural abundance, but also due to problems associated with their collection and re-collection [1]. In several cases, limitations on natural supplies require chemical synthesis to come into the scene as the structures elucidated by spectroscopic techniques need to be independently confirmed. Additionally, usually natural sources are not adequate for *in vivo* and structure-activity relationship studies [4].

**Figure 1:**
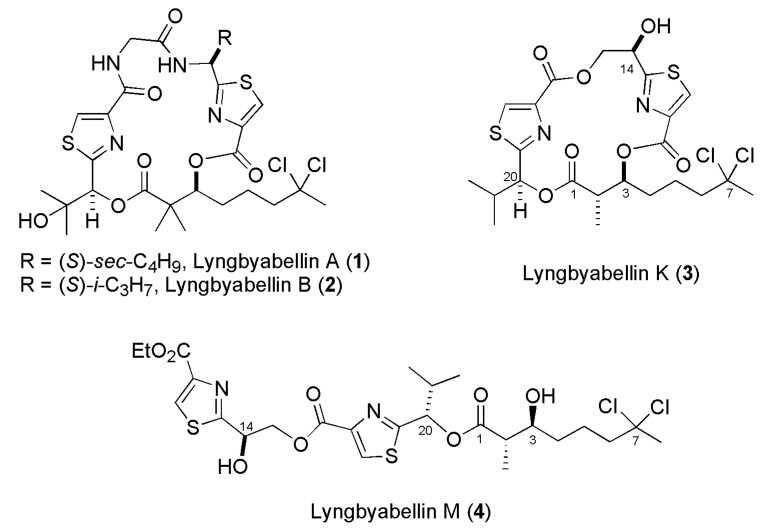

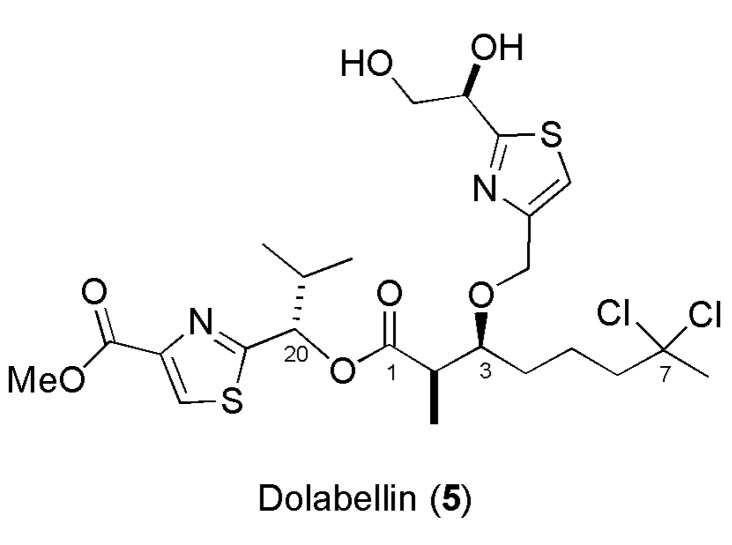
Chemical structures of lyngbyabellins A, B, K, M and Dolabellin.

The lyngbyabellins are a family of non-ribosomal peptide synthetase/polyketide synthase derived metabolites displaying thiazole rings and a distinct chlorinated octanoic acid chain produced by the genus *Moorea* sp. (formerly *Lyngbya* sp.). Lyngbyabellins A and B were isolated in 2000 from *Lyngbya majuscula* [5,6] and their structures have been confirmed by total synthesis (Figure 1) [7,8,9]. By now, 12 other lyngbyabellins have been reported from marine cyanobacteria. Recently, Choi and coworkers reported the isolation of five new representatives of the lyngbyabellin family which were named as lyngbyabellins K–N (**1** and **2**, respectively) from marine cyanobacterium *M. bouillonii* (PAL 8/3/09-1) [10].

Lyngbyabellin K (**3**) differs from lyngbyabellins A and B (**1** and **2**, respectively) as it does not contain the macrolactam/macrolactone ring displaying a bis macrolactone ring instead and it features a 2,3-*anti* relationship in the dichlorooctanoic acid moiety while lyngbyabellin M (**4**) corresponds to the open chain form (cleavage of the C-16-oxygen bond) of lyngbyabellin K (**3**). Dolabellin (**5**) is a cytotoxic bisthiazole metabolite isolated by Sone and coworkers from the Japanese sea hare *D. auricularia* with *syn* relative configuration in the dichlorooctanoic acid chain and opposite configuration at C-20 in comparison to lyngbyabellin M (**4**) [11,12,13]. At this juncture, it is still unclear whether lyngbyabellin M (**4**) is a natural compound or an isolation artifact. The relative configuration of lyngbyabellin K (**3**) was assigned based on spectroscopic data and its absolute configuration was established as 2*S*,3*S*,14*R*,20*S* from X-ray anomalous dispersion data. Lyngbyabellin M (**4**) was proposed to be of the same enantiomeric series as lyngbyabellin K (**3**) based on the similarities observed in the ^13^C-NMR shifts at most of the signals and similar optical rotation. Additionally, the configuration at C-2 and C-3 was confirmed by NMR analyses of the corresponding bis-Mosher esters and the absolute configuration of the hydroxy isovaleric residue was established after GC-MS comparison of the methyl ester obtained by hydrolysis of an authentic sample [10].

In this paper we present our synthetic approach to lyngbyabellin M (**4**) which allowed the confirmation of its 2*S*,3*S*,14*R*,20*S* configuration as proposed in the isolation studies.

## 2. Results and Discussion

**Scheme 1:**
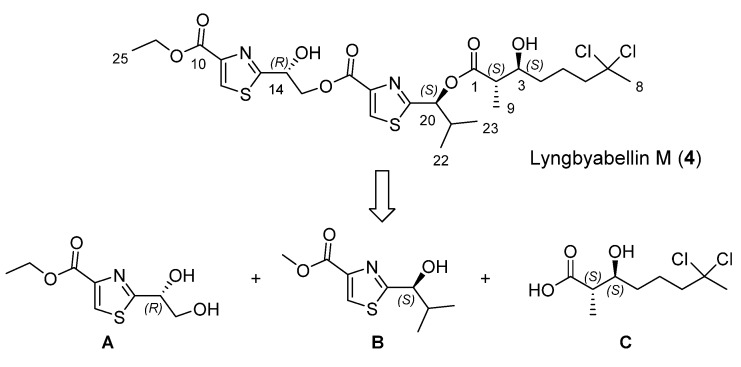
Retrosynthetic analysis for lyngbyabellin M (**4**).

Our approach to lyngbyabellin M (**4**) relies on the disconnection at the two ester bonds which simplifies its preparation to the synthesis of three chiral fragments **A**–**C** (Scheme 1).

**Scheme 2:**
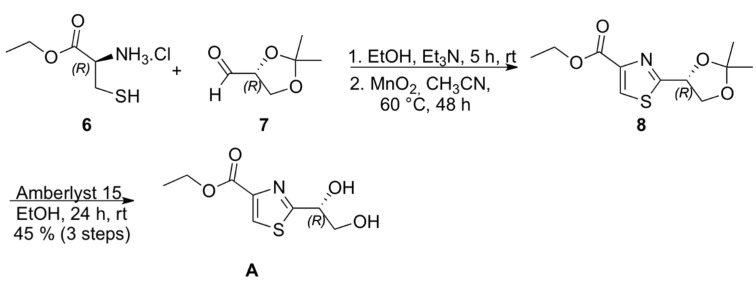
Synthesis of thiazole A.

Chiral thiazole **A** (Scheme 2) was prepared from condensation of (*S*)-cysteine ethyl ester hydrochloride (**6**) with (*R*)-isopropylidene glyceraldehyde (**7**), followed by MnO_2_ oxidation of the intermediate thiazolidine, according to the methodology described by Iwakawa and coworkers for the corresponding methyl ester, to afford 3,4-disubstituted thiazole **8** which underwent acetonide deprotection to provide thiazole **A** in 36% overall yield and specific optical rotation [α]_D_^20^ = +59 (MeOH, 1.15) [11,12,13].

**Scheme 3:**
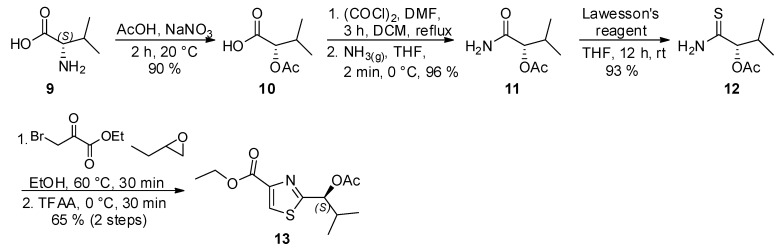
Synthesis of thiazole **13**.

As to the preparation of thiazole **B**, a modification of the Hantzch synthesis was employed to prepare **13**, following the procedure described by Schmidt and coworkers (Scheme 3). Accordingly, the reaction of thioamide **12**, readily obtained from (*S*)-valine (**9**), with ethyl bromopyruvate, followed by dehydration of the intermediate upon treatment with trifluoroacetic anhydride (TFAA) at 0 °C afforded thiazole **13** as white solid ([α]_D_^25^ = −37.0 (*c* 0.90, CHCl_3_); [α]_D_^25^ = −38.6 (*c* 1.09, CHCl_3_)) [14].

Methanolysis of thiazole **13** required a detailed investigation of the reaction conditions in order to avoid extensive racemization (Table 1). Initially, we carried out the hydrolysis/transesterification step according to the e procedure in the literature which provided the desired methyl ester in 70% yield but its optical rotation [α]_D_^25^ = −25.0 (*c* 0.65, CHCl_3_) was slightly lower than the one previously reported ([α]_D_^25^ = −31.2 (*c* 0.61,CHCl_3_)) [11,12,13]. Other hydrolytic conditions were tested and, in our hands, the best one proved to be the use of dibutyltin oxide in refluxing methanol [15] which afforded the desired fragment **B** in 71% yield and [α]_D_^25^ = −27.0 (*c* 0.70, CHCl_3_ ). Despite the less than optimal optical purity for intermediate **B**, we decided to move on in our synthetic plan as fragment **B** would be coupled with fragment **C** which was expected to be prepared in high enantiomeric ratio. In the event, the undesired diastereoisomer obtained after the coupling of fragments **B** and **C** would be removed by chromatography, affording the desired ester **25** in pure form.

**Table 1:** Hydrolysis conditions for the preparation of fragment **B**. 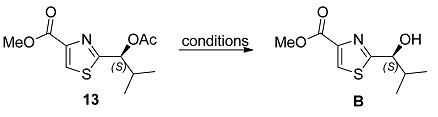

Entry	Conditions	Yield (%)	[α]_D_^25^ (*c*, CHCl_3_)
1	MeONa/MeOH, rt, 4 h	85	−25 (0.78)
2	K_2_CO_3_, MeOH, reflux, 2 h	70	−25 (0.65)
3	(i) LiOH.H_2_O, MeOH, rt, 1 h (ii) TMSCH_2_N_2_, THF, rt, 20 min	34	−20 (0.66)
4	Bu_2_SnO, MeOH, reflux, 12 h	71	−27 (0.78)

**Scheme 4:**
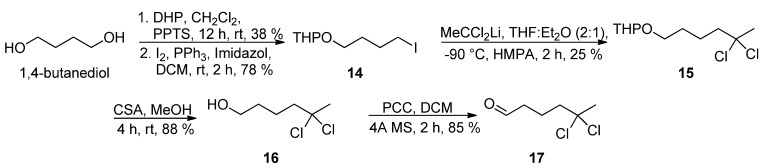
Synthesis of aldehyde **17**.

The preparation of *gem*-dichloro aldehyde **17** was initially investigated via the nucleophilic substitution reaction of iodide **14** [16,17] with the lithium anion derived from 1,1-dichloroethane to afford **15**, followed by removal of the tetrahydropyranyl group and oxidation of the primary alcohol in **16** (Scheme 4). However, this was not a practical solution as the *gem*-dichloro pyranyl ether **15** was obtained in 25% yield at most.

**Scheme 5:**
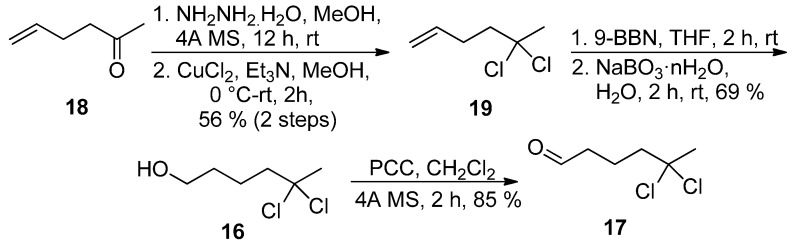
Alternative synthesis of aldehyde **17**.

The best solution came with the preparation of *gem*-dichloro alkene **19** in 56% yield (two steps) from ketone **18** using the oxidation of the corresponding hydrazone by CuCl_2_ as described by Takeda and coworkers [18]. Hydroboration-oxidation sequence, followed by oxidation of primary alcohol **16** secured preparation of *gem*-dichloro aldehyde **17** in 32% overall yield (Scheme 5).

**Scheme 6:**
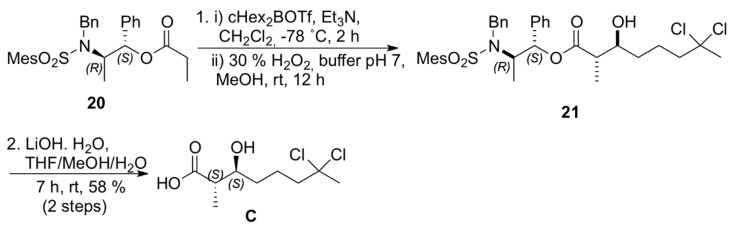
Synthesis of carboxilyc acid **C**.

The construction of carboxylic acid **C** was then accomplished using the *anti*-selective boron-mediated asymmetric aldol reaction with propionate **20** prepared from Masamune’s chiral auxiliary [19]. *anti-*Aldol **21** was prepared via enolization of ester with dicyclohexylboron triflate and triethylamine at −78 °C (Scheme 6) [20]. The absolute configuration at the two newly formed stereogenic centers was assigned after conversion of the aldol adduct **21** to the corresponding carboxylic acid **C** (58% yield for two steps) [21].

**Scheme 7:**
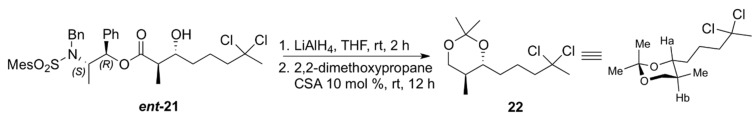
Conversion of *ent*-**21** to acetonide **22**.

Despite the previous results on the aldol reaction with propionate derived from Masamune’s auxiliary described in the literature [19], we decided to confirm the *anti* relative configuration via conversion of the carboxylic acid obtained from *ent*-**21** to the corresponding acetonide **22**, after reduction with LiAlH_4_ and treatment with 2,2-dimethoxypropane under acid catalysis (40% overall yield). The large coupling constant (^3^*J* = 11.6 Hz) observed between Ha and Hb in **22** revealed their *trans* disposition which translates to the expected *anti* configuration in the corresponding carboxylic acid (Scheme 7).

**Scheme 8:**
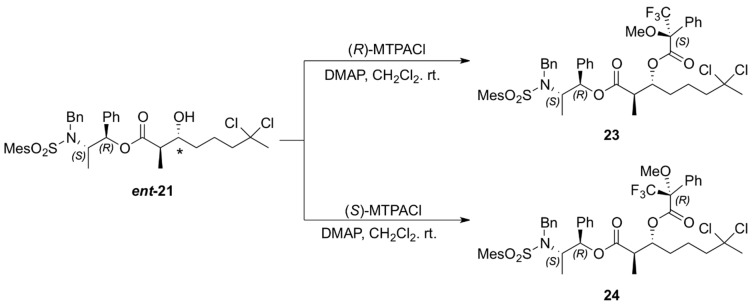
Preparation of the MTPA esters of *ent*-**21**.

The absolute configuration of the two newly generated stereogenic centers in *ent-***21** was assigned via the corresponding Mosher esters **23** and **24**, prepared from (*R*)- and (*S*)-α-methoxy-α- trifluoromethylphenylacetyl chloride (MTPACl), respectively (Scheme 8). Analyses of the ^1^H-NMR spectra for Mosher esters **23** and **24** revealed Δδ*_S_*_− *R*_ < 0 for H14, H15, H16 and H19 (up to −0.12 ppm) and positive values for H5 (+0.05 ppm) which translate to *R* configuration for both the carbinolic carbon and the methine carbon α to the carbonyl group in *ent*-**21** (Table 2) [22,23,24].

**Table 2:** Chemical shifts and Δδ*_S_*_−_ *_R_* values for MTPA esters derived from *ent*-**21**. 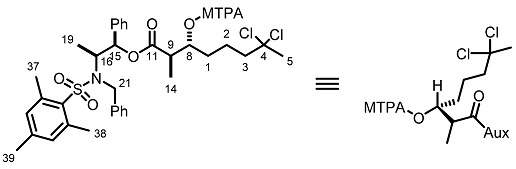

H	(*S*)-MTPA Ester (23)		(*R*)-MTPA Ester (24)		Δ (δ*S* − δ*R*)
	δH (ppm)	Multiplicity (*J*)	δH (ppm)	Multiplicity (*J*)	
H-5	1.98	*s*	1.93	*s*	+0.053
H-14	0.90	d (3H, *J* = 7.4)	1.02	d (3H, *J* = 7.2)	−0.12
H-15	5.73	d (1H, *J* = 5.1)	5.76	d (1H, *J* = 5.1)	−0.034
H-16	4.04	*m* (1H)	4.06	*m* (1H)	−0.02
H-19	1.07	d (3H, *J* = 6.9)	1.10	d (3H, *J* = 6.8)	−0.036
H-37/38	2.37	*s* (6H)	2.37	*s* (6H)	−0.025
H-39	2.20	*s* (3H)	2.21	*s* (3H)	−0.005

**Scheme 9:**
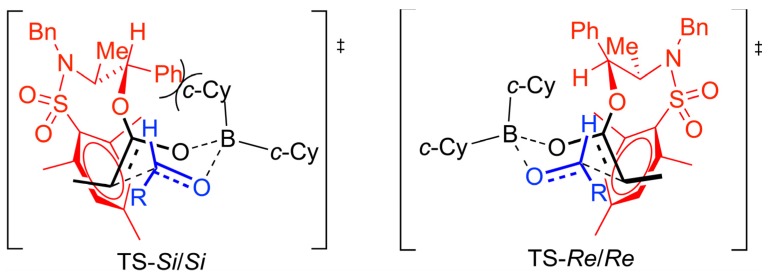
Proposed transition state model for the aldol reaction.

The preference for the *anti* stereochemistry observed for the major aldol product requires a kinetically controlled approach of the *E*-boron enolate *Re* face to the *Re* face of the aldehyde according to a 6-membered Zimmerman-Traxler transition state model. The stabilizing interaction involving the electron-deficient *N*-sulfonylmesityl group and the electron-rich double bond of the boron enolate can be accommodated in the transition state corresponding to the *Re*/*Re* topology while for the *Si*/*Si* topology which would lead to the diastereoisomeric *anti* aldol product, a destabilizing interaction develops between the pseudoaxial cyclohexyl group and the *N*-mesityl group of the *N*-mesitylsulfonyl norephedrine chiral auxiliary (Scheme 9).

**Scheme 10:**
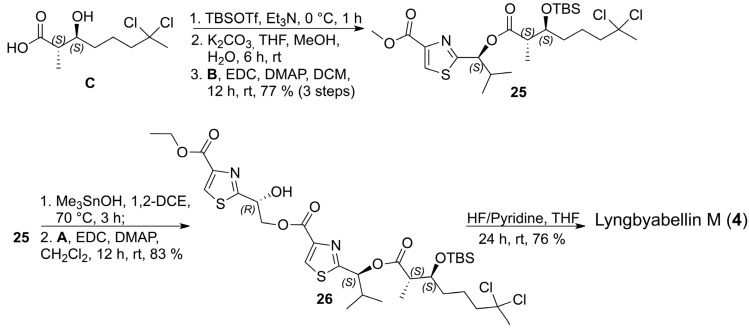
Final steps in the total synthesis of lyngbyabellin M (**4**).

With the three key fragments in hands, we initiated the assembly of the structure assigned for lyngbyabellin M (**4**) by coupling the carboxylic acid **C** with thiazole **B** which required the protection of the secondary alcohol in **C** as the corresponding *terc*-butyldimethylsilyl (TBS) ether, hydrolysis to the carboxylic acid, followed by coupling with thiazole **B**. After purification, the corresponding ester **25** was obtained in 77% yield as a single diastereoisomer over 3 steps, as indicated by the analysis of the^1^H- and ^13^C-NMR spectra. In order to install thiazole **A**, a chemoselective hydrolysis of the methyl ester in **25** was required which was accomplished using hydroxytrimethyltin in 1,2-dichloroethane without any indication of epimerization by ^1^H- and ^13^C-NMR analyses [25,26,27]. The corresponding carboxylic acid was prepared from **25** and it was coupled to thiazole **A** via the EDC/DMAP protocol to afford **26** in 83% yield over two steps (Scheme 10).

Comparison of the NMR data of the synthetic sample of lyngbyabellin M (**4**) prepared after deprotection of the TBS group in **26** with HF/pyridine with those available in the literature for lyngbyabellin M (**4**) revealed almost complete match except for C-13, C-14, C-15, C-16, C-20 and C-21. Personal information provided by Gerwick and coworkers after completion of our work provided revised ^13^C-NMR data for natural lyngbyabelin M (**4**) which nicely fit those obtained for the synthetic sample thus confirming the 2*S*,3*S*,14*R*,20*S* configuration proposed for the natural product by spectroscopic analysis and chemical correlation (Table 3).

**Table 3:** Comparison of the ^13^C-NMR data for natural (CDCl_3_, 125 MHz) and synthetic (CDCl_3_, 125 MHz) lyngbyabellin M.

Carbon	Natural Lyngbyabellin M *δ (ppm)	Synthetic Lyngbyabellin M δ (ppm)
**1**	174.4	174.3
**2**	44.4	44.4
**3**	75.4	75.3
**4**	34.4	34.4
**5**	21.5	21.5
**6**	50.0	49.9
**7**	90.8	90.8
**8**	37.7	37.6
**9**	15.0	14.9
**10**	161.8	161.7
**11**	147.6	147.5
**12**	128.2	128.2
**13**	171.2 (172.1) **	172.1
**14**	70.30 (70.05) **	70.0
**15**	70.26 (70.15) **	70.2
**16**	161.7 (160.6) *	160.5
**17**	145.9	145.7
**18**	129.2	129.1
**19**	170.5	170.4
**20**	77.5 (77.1) **	77.2
**21**	34.4 (34.2) **	34.3
**22**	19.5	19.4
**23**	16.8	16.7
**24**	61.8	61.7
**25**	14.7	14.6

* Spectroscopic data as appeared in [10]; ** Revised data by Gerwick and coworkers [28].

Although synthetic lyngbyabellin M (**4**) displayed the same absolute configuration as that originally proposed by Gerwick and coworkers for the natural product, the synthetic sample was shown to be dextrorotatory ([α]_D_^25^ = +12 (*c* = 0.5, MeOH)) while natural lyngbyabellin M was reported as levorotatory ([α]_D_^25^ = −4.5 (*c* =0.5, MeOH)). We are presently not able to explain this discrepancy since a sample of the natural product was not available for us to compare natural and synthetic lyngbyabellins M by chiral HPLC or other chiroptical techniques.

## 3. Experimental Section

Reagents and solvents were commercial grade and were used as supplied except when specified in the experimental procedure. Triethylamine and dichloromethane were distilled from calcium hydride and tetrahydrofuran was distilled from Na/benzophenone. Reactions were monitored by thin layer chromatography analysis using Merck Silica Gel 60 F-254 thin layer plates. Flash column chromatography was performed on Acros silica gel 60, 0.040–0.063 mm. ^1^H NMR and ^13^C NMR data were recorded on a Varian Inova (500 MHz for ^1^H and 125 MHz for ^13^C NMR) or Bruker Avance (250 MHz for ^1^H and 62.5 MHz for ^13^C NMR) spectrometers using as internal standard the residual nondeuterated solvent (CHCl_3_) or TMS (^1^H NMR). High resolution mass spectra (HRMS) for novel compounds were measured on a Waters XEVO Quadrupole-Time of Flight (Q-TOF) spectrometer (ESI) or in a Waters Premier (EI). Infrared spectra (IR) were obtained on iS5 spectrometer and absorptions are reported in reciprocal centimeters. Melting points were recorded on an Electrothermal 9100 melting point apparatus and were uncorrected.

(*R*)-Ethyl 2-(2,2-dimethyl-1,3-dioxolan-4-yl)thiazole-4-carboxylate (**8**). To a stirred solution of l-cysteine ethyl ester hydrochloride (4.28 g, 23 mmol) and triethylamine (3.37 mL, 1.1 equivalent) in ethanol (66 mL), a solution of freshly prepared (*R*)-(+)-glyceraldehyde acetonide [29,30] (2.86 g, 22 mmol) in ethanol (10 mL) was added. After stirring for 5 h at room temperature, the solvent was evaporated under reduced pressure. The crude product was dissolved in ether and washed with water (20 mL) and brine (15 mL). The organic phase was dried over MgSO_4_ and the solvent evaporated under reduced pressure. A colorless oil was obtained which was stirred in CH_3_CN (130 mL) in the presence of MnO_2_ (30.2 g, 20 equiv.) at 60 °C. The reaction progression was followed by TLC and after 48 h the crude mixture was filtered over Celite. Solvent evaporation under reduced pressure was followed by purification by column chromatography on silica gel, with hexanes–ethyl acetate (7:3) mixture as the eluent. An orange oil was obtained (2.951 g, 11.44 mmol) in 52% yield. IR (cm^−1^, ATR): 2986, 1719, 1372, 1208; ^1^H NMR (250 MHz, CDCl_3_): δ 8.19 (s, 1H), 5,43 (dd, 1H, *J* = 6.7 and 5.1 Hz), 4.52-4.36 (m, 3H), 4.10 (dd, 1H, *J* = 8.8 and 5.1 Hz), 1.58 (s, 3H), 1.46 (s, 3H), 1.40 (t, 3H, *J* = 7.1 Hz); ^13^C NMR (62.5 MHz, CDCl_3_): δ 172.8, 160.5, 146.7, 126.9, 110.4, 74.7, 69.6, 60.6, 25.7, 24.4, 13.7. HRMS (ESI/+, *m*/*z*): Calcd for: C_11_H_15_NO_4_S, 258.0800; found: 258.0804; [α]_D_^25^ = +46 (CH_2_Cl_2_, 1.07).

(*R*)-Ethyl 2-(1,2-dihydroxyethyl)thiazole-4-carboxylate (**A**). To a stirred solution of ester **8** (2.01 g, 7.8 mmol) in ethanol (30 mL), Amberlist 15 (10% m/m, 0.20 g) was added. The reaction progression was followed by TLC and after 3 h, the mixture was filtered and the solvent was evaporated under reduced pressure. The crude product was recrystallized from hexanes–ethyl acetate (2:1) and a white solid (1.19 g, 5.46 mmol) was obtained in 70% yield. MP 152–153 °C; IR (cm^−1^, ATR): 3293, 3114, 1716, 1507, 1234, 1052; ^1^H NMR (250 MHz, MeOD): δ 8.32 (s, 1H), 4.96 (dd, 1H, *J* = 6.0 and 3.8 Hz), 4.37 (q, 2H, *J* = 7.1 Hz), 3.93 (dd, 1H, *J* = 11.5 and 3.8 Hz), 3.76 (dd, 1H, *J* = 11.5 and 6.0 Hz), 1.38 (t, 3H, *J* = 7.1 Hz); ^13^C NMR (62.5 MHz, MeOD): δ 177.2, 162.8, 147.7, 129.2, 73.6, 67.2, 62.4, 14.6. HRMS (ESI/+, *m*/*z*): Calcd: C_8_H_11_NO_4_S, 218.0487; Found: 218.0475; [α]_D_^25^ = +59 (MeOH, 1.15).

(*S*)-Methyl 2-(1-hydroxy-2-methylpropyl)thiazole-4-carboxylate (Thiazole **B**). To a solution of thiazol **13**^14^ (0.503 g, 1.85 mmol) in anhydrous methanol (25 mL) was added dibutyltinoxide (2.30 g, 9.38 mmol). The suspension was stirred 12 h under reflux and the reaction course was monitored by TLC. The solids were removed by filtration and the solvent was removed under reduced pressure. The crude product was purified by flash chromatography in silica gel using an isocratic mixture of hexanes-ethyl acetate (7:3, V/V). A colorless solid was isolated in 71% yield (0.283 g, 1.31 mmol). MP 50–52 °C; IR (cm^−1^, ATR): 3279, 2968, 1735, 1714, 1212. ^1^H NMR (250 MHz, CDCl_3_): δ 8.11 (s, 1H), 4.86 (d, *J* = 4.7 Hz, 1H), 3.90 (s, 3H), 3.36 (br s, 1H), 2.29–2.15 (m, 1H), 0.98 (d, *J* = 6.8 Hz, 3H), 0.90 (d, *J* = 6.8 Hz, 3H). ^13^C NMR (62.5 MHz, CDCl_3_): δ 176.2, 161.9, 146.2, 127.5, 76.5, 52.4, 34.9, 18.8, 16.2. HRMS (ESI/+, *m*/*z*): Calcd: C_9_H_14_NO_3_S, 216.0694; Found: 216.0703. [α]_D_^25^ = −27 (CHCl_3_, 0.78).

5,5-Dichlorohexan-1-ol (**16**). To a flask containing 4Å molecular sieves (15 g) was added methanol (70 mL) and hydrazine monohydrate (5 mL, 5 equiv.) were added successively with stirring. After 20 min, 5-hexen-2-one (**18**, 2.4 mL, 20.4 mmol) was added dropwise to the reaction mixture and the mixture was stirred overnight. Molecular sieves were filtered off and washed with ethanol. The combined filtrate was concentrated under reduced pressure and the excess hydrazine was further removed by azeotropic distillation with ethanol under reduced pressure. The crude product was dissolved in methanol (60 mL). A solution of anhydrous copper(II) chloride (12.1 g, 6 equiv.) and triethylamine (8.5 mL, 3 equiv.) in methanol (120 mL) was prepared after stirring for 10 min. at 20 °C and cooled to 0 °C. To this solution was added dropwise the above hydrazone solution over 15 min. The cooling bath was removed and stirring was continued for 2 h. The reaction mixture was quenched by the addition of aqueous ammonia solution (3.5% v/v, 10 mL) and the mixture was extracted with cold pentane (3 × 150 mL), washed with brine, dried (Na_2_SO_4_), and carefully concentrated under reduced pressure. A volatile colorless oil (1.74 g, 11.4 mmol) was obtained in 56% crude yield. ^1^H NMR (250 MHz, CDCl_3_): δ 5.94–5.74 (m, 1H), 5.16–4.95 (m, 2H), 2.50-2.36 (m, 2H), 2.35–2.25 (m, 2H), 2.17 (s, 3H); ^13^C NMR (62.5 MHz, CDCl_3_): δ 136.4, 115.6, 90.1, 48.8, 37.4, 29.9. A round bottomed flask equipped with a nitrogen inlet containing the colorless oil above, 9-borabicylcononane solution (0.5 M in THF, 24 mL, 11.6 mmol) was added via syringe. After stirring 2 h at room temperature, water (10 mL) was added, followed by sodium perborate (4.3 g, 3 equiv.). The mixture was vigorously stirred for 2 h and extracted with diethyl ether. The combined organic phase was washed with saturated NaCl solution (10 mL) and dried (MgSO_4_), and the product was purified by column chromatography on silica gel using hexanes–ethyl acetate (6:4) mixture as the eluent. A colorless oil (1.325 g, 7.75 mmol) was obtained in 38% overall yield from 18. IR (cm^−1^, ATR): 3345, 2936, 2873, 1440, 1380, 1169, 1071, 1050; ^1^H NMR (250 MHz, CDCl_3_): δ 3.60 (t, 2H, *J* = 6.3 Hz), 2.82 (br s, 1H), 2.23–2.13 (m, 2H), 2.10 (s, 3H), 1.75–1.49 (m, 4H); ^13^C NMR (62.5 MHz, CDCl_3_): δ 90.7, 62.2, 49.5, 37.3, 31.8, 22.1.

5,5-Dichlorohexanal (**17**). To a mixture of pyridinium chlorochromate (0.97 g, 2 equiv.) and 4Å molecular sieves (1 g) in DCM (40 mL) was added a solution of the alcohol **16** (0.38 g, 2.2 mmol) in DCM (5 mL). The mixture was then stirred at room temperature for 1 h and the residue was filtered through a short pad of Florisil and silica gel, washed with DCM and the solvent was evaporated under reduced pressure. A volatile colorless oil (0.82 g, 85% crude yield) was obtained and used without purification in the next step. IR (cm^−1^, ATR): 2932, 2726, 1723, 1448, 1381, 1171; ^1^H NMR (250 MHz, CDCl_3_): δ 9.78 (t, 1H, *J* = 1.3 Hz), 2.54 (td, 2H, *J* = 7.1 and 1.3 Hz), 2.26–2.18 (m, 2H), 2.15 (s, 3H), 2.07–1.93 (m, 2H); ^13^C NMR (62.5 MHz, CDCl_3_): δ 201.3, 90.0, 48.7, 42.8, 37.3, 18.3.

(2*S*,3*S*)-7,7-Dichloro-3-hydroxy-2-methyloctanoic acid (**C**). To an oven-dried round-bottomed flask, a stirred solution of (1*S*,2*R*)-**20** (0.500 g, 1.04 mmol) and triethylamine (0.35 mL, 2.4 equiv.) in DCM (5 mL) was added and cooled to −78 °C. A solution of freshly prepared *c*-Hex_2_BOTf (1.0 M in hexane, 2.3 mL, 2.2 equiv.) was added dropwise via syringe over 15 min. The resulting solution was stirred at −78 °C for 30 min. and a solution of aldehyde **17** (0.21 g, 1.2 equiv.) in DCM (2 mL) was added dropwise. The reaction mixture was stirred at −78 °C for 2 h and allowed to warm to room temperature over 1 h. Then, the reaction mixture was quenched by the addition of phosphate buffer solution (pH 7, 5 mL), followed by MeOH (15 mL) and 30% aqueous H_2_O_2_ (2 mL, careful addition). The mixture was vigorously stirred overnight and then concentrated under reduced pressure. The residue was partitioned between water (15 mL) and DCM (30 mL). The aqueous layer was extracted with DCM and the combined organic layer was concentrated. The aldol product was isolated by chromatography on silica gel using hexanes-ethyl acetate (8:2) as eluent. A viscous colorless oil containing a small quantity of cyclohexanol was dissolved in a water-MeOH-THF solution (1:1:1, 9 mL) and LiOH·H_2_O (126 mg, 5 equiv.) was added. The mixture was stirred overnight at room temperature and poured into water (10 mL). After extraction with ether to recover the chiral auxiliary, the aqueous layer was acidified with 1M HCl to pH 2 and extracted with DCM. The organic layer was washed with brine and dried with Na_2_SO_4_. After solvent concentration under reduced pressure, the product was purified by chromatography on a short pad of silica gel with gradient. The nonpolar products were removed with hexanes-ethyl acetate (7:3) and the acid **C** was removed with ethyl acetate to give a viscous colorless oil (0.145 g, 0.60 mmol) in 58% yield over two steps. IR (cm^−1^, ATR): 3344, 2984, 2964, 1694, 1208; ^1^H NMR (250 MHz, CDCl_3_): δ 3.65–3.85 (m, 1H), 2.57 (qt, 1H, *J* = 7.1), 2.28–2.18 (m, 2H), 2.14 (s, 3H), 1.96–1.48 (m, 4H), 1.24 (d, 3H, *J* = 7.1); ^13^C NMR (62.5 MHz, CDCl_3_): δ 180.8, 90.5, 73.0, 49.5, 45.3, 37.3, 33.6, 21.9, 14.1. HRMS (ESI/−, *m*/*z*): Calcd: C_9_H_16_Cl_2_O_3_, 241.0398; Found: 241.0439. [α]_D_^25^ = −6 (MeOH, 1.00).

Methyl-2-((*S*)-1-(((2*S*,3*S*)-3-((*tert*-butyldimethylsilyl)oxy)-7,7-dichloro-2-methyloctanoyl)oxy)-2-methylpropyl)thiazole-4-carboxylate (**25**). Carboxylic acid **C** (0.14 g, 0.60 mmol) was dissolved in DCM (3 mL) and cooled to 0 °C. To this solution, triethylamine (0.5 mL, 6 equiv.) and *tert-*butyldimethylsilyl triflate (0.36 mL, 2.6 equiv.) were added. The mixture was stirred for 2 h at 0 °C. Then potassium carbonate (0.41 g, 5.85 mmol), water (4 mL), MeOH (4 mL), and THF (4 mL) were added, and the mixture was stirred for 6 h at room temperature. To this solution ethyl acetate (20 mL) and brine (5 mL) were added and the mixture was acidified with 1M HCl (pH 2). The organic layer was separated, and the aqueous layer was extracted with ethyl acetate (2 × 20 mL). The combined organic layers were dried (Na_2_SO_4_), and concentrated under reduced pressure. The remaining oil (0.21 g) was dissolved in DCM (3 mL) and EDC (0.13 g, 1.2 equiv.), thiazole **B** (0.14 g, 1.1 equiv.) and catalytic quantity of DMAP were added. The mixture was stirred overnight at room temperature and quenched by addition of brine (5 mL), followed by extraction with ethyl acetate (3 × 20 mL). The organic layer was dried with Na_2_SO_4_ and after solvent evaporation under reduced pressure, the crude product was purified by chromatography on silica gel using hexanes-ethyl acetate (7:3). A colorless oil (0.256 g, 0.462 mmol) was obtained in 77% yield for two steps. IR (ATR, cm^−1^): 2956, 2931, 2856, 1744, 1244, 1107; ^1^H NMR (250 MHz, CDCl_3_): δ 8.12 (s, 1H), 6.03 (d, 1H, *J* = 5.3 Hz), 4.13–4.03 (m, 1H), 3.92 (s, 3H), 2.86–2.71 (m, 1H), 2.48–2.33 (m, 1H), 2.10 (s, 3H), 1.84–1.12 (m, 9H), 0.95 (s, 3H), 0.93 (s, 3H), 0.83 (s, 9H), 0.07 (s, 3H), 0.01 (s, 3H); ^13^C NMR (62.5 MHz, CDCl_3_): δ 172.9, 170.5, 161.7, 146.9, 127.2, 90.5, 77.1, 72.6, 52.4, 49.7, 45.5, 37.3, 33.5, 32.1, 25.7, 21.7, 18.6, 17.9, 17.0, 10.9, −4.6, −4.7. HRMS (ESI/+, *m*/*z*): Calcd: C_24_H_42_Cl_2_NO_5_SSi, 554.1930; Found: 554.1943. [α]_D_^25^ = −10 (CH_2_Cl_2_, 0.8).

(*R*)-2-(4-(Ethoxycarbonyl)thiazol-2-yl)-2-hydroxyethyl-2-((*S*)-1-(((2*S*,3*S*)-3-((*tert*-butyldimethylsilyl)oxy)-7,7-dichloro-2-methyloctanoyl)oxy)-2-methylpropyl)thiazole-4-carboxylate (**26**). Carboxylic ester **25** (88.7 mg, 0.16 mmol) was dissolved in 1,2-dichloroethane (3 mL) and after addition of trimethyltin hydroxide (0.18 g, 6 equiv.), the mixture was heated at 70 °C until the reaction was complete by TLC analysis. After 2 h, the mixture was concentrated *in vacuo*, and the residue was taken up in ethyl acetate (15 mL). The organic layer was washed successively with HCl (5% v/v, 5 mL) and brine (5 mL) and dried over sodium sulfate. Removal of the solvent *in vacuo* afforded the corresponding carboxylic acid as colorless oil (73 mg) which was dissolved in DCM (2 mL). To this solution EDC (17 mg, 1.1 equiv.), compound **A** (17 mg, 1.1 equiv.) and catalytic quantity of DMAP were added. The mixture was stirred overnight at room temperature and quenched by addition of brine (5 mL), followed by extraction with ethyl acetate (3 × 20 mL). The organic layer was dried with Na_2_SO_4_ and after solvent concentration, the product was purified by preparative thin layer chromatography on silica gel using chloroform-ethyl acetate (7:3). A colorless oil (0.0982 g, 0.133 mmol) was obtained in 83% yield. IR (ATR, cm^−1^): 2956, 2931, 2856, 1744, 1244, 1107; ^1^H NMR (250 MHz, CDCl_3_): δ 8.17 (s, 2H), 5.98 (d, 1H, *J* = 5.6 Hz), 5.47 (dd, 1H, *J* = 6.9 and 3.2 Hz), 4.88 (dd, 1H, *J* = 11.5 and 3.2 Hz), 4.62 (dd, 1H, *J* = 11.5 and 7.4 Hz), 4,41 (q, 2H, *J* = 7.1 Hz), 4.12–4.04 (m, 1H), 2.83–2.76 (m, 1H), 2.43–2.34 (m, 1H), 2.13 (s, 3H), 1.83–1.49 (m, 4H), 1.40 (t, 3H, *J* = 7.2 Hz), 1.18 (d, 3H, *J* = 7.0 Hz), 0.96 (dd, 6H, *J* = 6.2 Hz), 0.84 (s, 9H), 0.08 (s, 3H), 0.01 (s, 3H); ^13^C NMR (62.5 MHz, CDCl_3_): δ 173.0, 171.9, 170.9, 161.3, 161.1, 147.2, 146.0, 128.3, 127.9, 90.6, 77.0, 72.7, 70.5, 68.9, 61.5, 49.7, 45.4, 37.4, 33.5, 32.2, 29.6, 25.7, 21.6, 18.6, 17.9, 17.2, 14.3, 11.0, −4.6, −4.7. HRMS (ESI/+. *m*/*z*): Calcd: C_31_H_49_Cl_2_N_2_O_8_S_2_Si. 739.2076; Found: 739.2107; [α]_D_^25^ = +23 (CHCl_3_, 0.65).

(*R*)-2-(4-(Ethoxycarbonyl)thiazol-2-yl)-2-hydroxyethyl 2-((*S*)-1-(((2*S*,3*S*)-7,7-dichloro-3-hydroxy-2-methyloctanoyl)oxy)-2-methylpropyl)thiazole-4-carboxylate (**4**). A teflon test tube was charged with a solution of compound **26** (0.0210 g, 0.0284 mmol) in THF (1 mL) and a solution of pyridine-HF (70%, 0.15 mL, 250 equiv.) was added. The solution was stirred for 24 h at room temperature and the reaction mixture was dissolved in ethyl acetate (20 mL), washed with water (5 mL), brine (5 mL) and dried over Na_2_SO_4_. After solvent concentration under reduced pressure, the product was purified by preparative thin layer chromatography on silica gel using chloroform–ethyl acetate (6:4). A colorless oil (0.0135 g, 0.0216 mmol) was obtained in 76% yield. IR (ATR, cm^−1^): 3337, 3118, 2967, 2935, 1731, 1485, 1325, 1210, 1170, 1099; ^1^H NMR (500 MHz, CDCl_3_): δ 8.30 (s, 1H), 8.18 (s, 1H), 5.99 (d, 1H, *J* = 3.7 Hz), 5.42 (dd, 1H, *J* = 9.1 and 2.5 Hz), 4.95 (dd, 1H, *J* = 11.3 and 2.5 Hz), 4.43 (q, 2H, *J* = 7.1 Hz), 4.31 (dd, 1H, *J* = 11.3 and 9.0 Hz), 3.81 (q, 2H, *J* = 5.1 Hz), 2.89–2.81 (m, 1H), 2.39–2.19 (m, 4H), 2.16 (s, 3H), 1.96–1.83 (m, 4H), 1.41 (t, 3H, *J* = 7.1 Hz), 1.26 (d, 4H, *J* = 7.1 Hz), 1.15 (d, 3H, *J* = 6.7 Hz), 1.00 (d, 3H, *J* = 7.1 Hz); ^13^C NMR (125 MHz, CDCl_3_): δ 174.4, 172.1, 170.5, 161.7, 160.5, 147.5, 145.7, 129.1, 128.2, 90.8, 75.4, 70.2, 70.2, 61.7, 49.9, 37.7, 34.5, 34.3, 21.5, 19.4, 16.7, 15.0, 14.6. HRMS (ESI/+. *m*/*z*): Calcd: C_25_H_35_Cl_2_N_2_O_8_S_2_: 625.1212, Found: 625.1185, [α]_D_^25^ = +12 (MeOH, 0.5).

## 4. Conclusions

The first total synthesis of (+)-lyngbyabellin M (**4**) was described involving the coupling of two chiral thiazole intermediates and a chiral carboxylic acid stereoselectively prepared in three steps from commercially available Masamune’s chiral auxiliary via an *anti* selective aldol reaction. A transition state model to rationalize the *anti* preference was proposed based on the preferential formation of and (*E*)-boron enolate and the intervenience of a Zimmerman-Traxler approach to minimize steric repulsion between the *N*-sulfonylmesityl group in the chiral auxiliary and the cyclohexyl groups of the boron enolate in the transition state. (+)-Lyngbyabellin M (**4**) was prepared in 28% overall yield from commercially available Masamune’s chiral auxiliary (1*S*,2*R*)-propionate **20**, and its spectroscopy identity to natural lyngbyabellin M was confirmed by ^1^H- and ^13^C NMR spectroscopies. The difference observed in the specific optical rotation of synthetic and natural lyngbyabellin M was assigned to the presence of levorotatory impurities in the natural sample.

## References

[1] Gerwick W.H., Moore B.S. (2012). Lessons from the past and charting the future of marine natural products drug discovery and chemical biology. Chem. Biol..

[2] Tan L.T. (2007). Bioactive natural products from marine cyanobacteria for drug discovery. Phytochemistry.

[3] Tan L.T. (2010). Filamentous tropical marine cyanobacteria: A rich source of natural products for anticancer drug discovery. J. Appl. Phycol..

[4] Hamada Y., Shioiri T. (2005). Recent progress of the synthetic studies of biologically active marine cyclic peptides and depsipeptides. Chem. Rev..

[5] Luesch H., Yoshida W.Y., Moore R.E., Paul V.J., Mooberry S.L. (2000). Isolation, structure determination, and biological activity of lyngbyabellin A from the marine cyanobacterium *Lyngbya majuscula*. J. Nat. Prod..

[6] Milligan K.E., Marquez B.L., Williamson R.T., Gerwick W.H. (2000). Lyngbyabellin B, a toxic and antifungal secondary metabolite from the marine cyanobacterium *Lyngbya majuscula*. J. Nat. Prod..

[7] Yokokawa F., Sameshima H., Shioiri T. (2001). Total synthesis of lyngbyabellin A, a potent cytotoxic metabolite from the marine cyanobacterium *Lyngbya majuscula*. Tetrahedron Lett..

[8] Yokokawa F., Sameshima H., Katagiri D., Aoyama T., Shioiri T. (2002). Total syntheses of lyngbyabellins A and B, potent cytotoxic lipopeptides from the marine cyanobacterium *Lyngbya majuscula*. Tetrahedron.

[9] Pang H., Xu Z., Chen Z., Ye T. (2005). Total synthesis of lyngbyabellin A. Lett. Org. Chem..

[10] Choi H., Mevers E., Byrum T., Valeriote F.A., Gerwick W.H. (2012). Lyngbyabellins K–N from two Palmyra Atoll collections of the marine cyanobacterium *Moorea bouillonii*. Eur. J. Org. Chem..

[11] Du J., Qu F., Lee D., Newton M.G., Chu C.K. (1995). 1,3-Dioxolane C-nucleosides: Asymmetric syntheses of four stereoisomers of 2-(2-hydroxymethyl0-1,3-dioxolan-5-yl))-1,3-thiazole-4-carboxamide. Tetrahedron Lett..

[12] Iwakawa M., Kobayashi Y., Ikuta S., Yoshimura J. (1982). A facile synthetic approach to the fragment D of antibiotic nosiheptide, 2-(1-amino-3-carboxy-3-hydroxy-(1*S*,3*S*)-propyl)-thiazole-4-carboxylic acid. Chem. Lett..

[13] Sone H., Kondo T., Kiryu M., Ishiwata H., Ojika M., Yamada K. (1995). Dolabellin, a cytotoxic bisthiazole metabolite from the sea hare *Dolabella auricularia*: Structural determination and synthesis. J. Org. Chem..

[14] Schmidt U., Gleich P., Griesser H., Utz R. (1986). Amino Acids and Peptides. 58. Synthesis of optically active 2-(1-hydroxyalkyl)-thiazole-4-carboxylic acids and 2-(1-aminoalkyl)-thiazole-4-carboxylic acids. Synthesis.

[15] Baumhof P., Mazitschek R., Giannis A. (2001). A mild and effective method for the transesterification of carboxylic acid esters. Angew. Chem. Int. Ed..

[16] Kato M., Kageyama M., Tanaka R., Kuwahara K., Yoshikoshi A. (1975). Synthetic study of (+/−)-canadensolide and related dilactones. Double lactonization of unsaturated dicarboxylic acids via acyl hypoiodite intermediates. J. Org. Chem..

[17] Liblikas I., Mozūraitis R., Santangelo E.M., Noreika R., Borg-Karlson A.-K. (2009). Syntheses, characterizations, and biological activities of tetradeca-4,8-dien-1-yl acetates as sex attractants of leaf-mining moth of the genus *Phyllonorycter* (Lepidoptera: Gracillariidae). Chem. Biodivers..

[18] Takeda T., Sasaki R., Yamauchi S., Fujiwara T. (1997). Transformation of ketones and aldehydes to gem-dihalides via hydrazones using copper(II) halides. Tetrahedron.

[19] Abiko A., Liu J.-F., Masamune S. (1997). The anti-selective boron-mediated asymmetric aldol reaction of carboxylic esters. J. Am. Chem. Soc..

[B20-marinedrugs-13-03309] 20.The diastereoisomeric ratio in the aldol reaction was determined by ^1^H-NMR analysis of the corresponding Mosher esters during studies for the assignement of the absolute configuration of the major diastereoisomer *ent*-**21**.

[21] Cetusic J.R.P., Green F.R., Graupner P.R., Oliver M.P. (2002). Total synthesis of hectochlorin. Org. Lett..

[22] Dale J.A., Mosher H.S. (1973). Nuclear magnetic resonance enantiomer reagents. Configurational correlations via nuclear magnetic resonance chemical shifts of diastereomeric mandelate, *O*-methylmandelate, and alpha-methoxy-alpha-trifluoromethylphenylacetate (MTPA) esters. J. Am. Chem. Soc..

[23] Seco J.M., Quiñoá E., Riguera R. (2004). The assignement of absolute configuration by NMR. Chem. Rev..

[24] Seco J.M., Quiñoá E., Riguera R. (2000). The assignement of absolute configurations by NMR of arylmethoxyacetate derivatives: Is this methodology being correctly used?. Tetrahedron Asym..

[25] Furlan R.L.E., Mata E.G., Mascaretti O.A. (1996). Cleavage of carboxylic esters effected by organotin oxides and hydroxides under classical heating and microwave irradiation. A comparative study. Tetrahedron Lett..

[26] Furlan R.L.E., Mata E.G., Mascaretti O.A., Pena C., Coba M.P. (1998). Selective detachment of Boc-protected amino acids and peptides from Merrifield, PAM and Wang resins by trimethyltin hydroxide. Tetrahedron.

[27] Nicolaou K.C., Estrada A.A., Zak M., Lee S.H., Safina B.S. (2005). A mild and selective method for the hydrolysis of esters with trimethyltin hydroxide. Angew. Chem. Int. Ed..

[28] Gerwick W.H. (2013). Personal communication.

[29] Debost J.L., Gelas J., Horton D. (1983). Selective preparation of mono- and diacetals of d-mannitol. J. Org. Chem..

[30] Jackson D.Y. (1988). An improved preparation of (+)2,3-*O*-isopropylidene-d-glyceraldehyde. Synth. Commun..

